# Supplementary material for: Calponin-homology domain mediated bending of membrane-associated actin filaments
Source: eLife. 2021 Jul 16;10:e61078. doi: 10.7554/eLife.61078 (PMC8315802; doi:10.7554/eLife.61078)
Supplement: Supplementary file 2. [file elife-61078-supp2.docx]

**Table 1: Plasmids used in this study**

| **Plasmid name** | **Amino acid sequence** | **MW /kDa** |
| --- | --- | --- |
| pET28C-6HIS-Rng2(1-189) | MGSSHHHHHHSSGLVPRGSHMASMTGGQQMGRIPMDVNVGLSRLQSQAGAPVGTKGSNTRLAAKQRETLQAYDYLCRVDEAKKWIEECLGTDLGPTSTFEQSLRNGVVLALLVQKFQPDKLIKIFYSNELQFRHSDNINKFLDFIHGIGLPEIFHFELTDIYEGKNLPKVIYCIHALSYFLSMQDLAPPLIKSDENLSFTDEDVSIIVRRLRQSNVILPNFKA | 25 |
| pET28C-6HIS-Rng2(1-250) | MGSSHHHHHHSSGLVPRGSHMASMTGGQQMGRIPMDVNVGLSRLQSQAGAPVGTKGSNTRLAAKQRETLQAYDYLCRVDEAKKWIEECLGTDLGPTSTFEQSLRNGVVLALLVQKFQPDKLIKIFYSNELQFRHSDNINKFLDFIHGIGLPEIFHFELTDIYEGKNLPKVIYCIHALSYFLSMQDLAPPLIKSDENLSFTDEDVSIIVRRLRQSNVILPNFKALSADFMLRASPVSSRTPSPTRFPKHARFQTLNSSDSASIYSSPYTSPTLEFSKKDASARSD | 32 |
| pET28C-6HIS-Rng2(1-300) | MGSSHHHHHHSSGLVPRGSHMASMTGGQQMGRIPMDVNVGLSRLQSQAGAPVGTKGSNTRLAAKQRETLQAYDYLCRVDEAKKWIEECLGTDLGPTSTFEQSLRNGVVLALLVQKFQPDKLIKIFYSNELQFRHSDNINKFLDFIHGIGLPEIFHFELTDIYEGKNLPKVIYCIHALSYFLSMQDLAPPLIKSDENLSFTDEDVSIIVRRLRQSNVILPNFKALSADFMLRASPVSSRTPSPTRFPKHARFQTLNSSDSASIYSSPYTSPTLEFSKKDASARSDILKMHRRTKSATPSLEQFNEPYKQTLPSHSIEFEDSFFQPPSQKGHMQRS | 38.5 |
| pETMCN-Rng2(1-189)-C-6HIS | MDVNVGLSRLQSQAGAPVGTKGSNTRLAAKQRETLQAYDYLCRVDEAKKWIEECLGTDLGPTSTFEQSLRNGVVLALLVQKFQPDKLIKIFYSNELQFRHSDNINKFLDFIHGIGLPEIFHFELTDIYEGKNLPKVIYCIHALSYFLSMQDLAPPLIKSDENLSFTDEDVSIIVRRLRQSNVILPNFKAGSHHHHHH | 25 |
| pET23a-10HIS-SNAP-Rng2(1-300) | MEEHHHHHHHHHHGSLEVLFQGPLGSMDKDCEMKRTTLDSPLGKLELSGCEQGLHRIIFLGKGTSAADAVEVPAPAAVLGGPEPLMQATAWLNAYFHQPEAIEEFPVPALHHPVFQQESFTRQVLWKLLKVVKFGEVISYSHLAALAGNPAATAAVKTALSGNPVPILIPCHRVVQGDLDVGGYEGGLAVKEWLLAHEGHRLGKPGLGGTGGSGTGGSMDVNVGLSRLQSQAGAPVGTKGSNTRLAAKQRETLQAYDYLCRVDEAKKWIEECLGTDLGPTSTFEQSLRNGVVLALLVQKFQPDKLIKIFYSNELQFRHSDNINKFLDFIHGIGLPEIFHFELTDIYEGKNLPKVIYCIHALSYFLSMQDLAPPLIKSDENLSFTDEDVSIIVRRLRQSNVILPNFKALSADFMLRASPVSSRTPSPTRFPKHARFQTLNSSDSASIYSSPYTSPTLEFSKKDASARSDILKMHRRTKSATPSLEQFNEPYKQTLPSHSIEFEDSFFQPPSQKGHMQRS | 57 |
| pET28C-6HIS-ScIqg1 (1-330) | MGSSHHHHHHSSGLVPRGSHMASMTGGQQMGRIPMTAYSGSPSKPGNNNSYLNRYVENLGTNVTPPLRPQSSSKINSSLNIASPSHLKTKTSASNSSATILSKKVESSVSKLKPSLPNKLVGKYTVDLSNYSKIELRYYEFLCRVSEVKIWIEAVIEEALPSEIELCVGDSLRNGVFLAKLTQRINPDLTTVIFPAGDKLQFKHTQNINAFFGLVEHVGVPDSFRFELQDLYNKKNIPQVFETLHILISMINKKWPGKTPALTNVSGQISFTKEEIAACKKAWPRIRDFKSLGTNINTAPASPEEPKEKRSGLIKDFNKFERPNIPVEEILITPRKNITDANCSDFSNTPSPYNEAPKMSNLDV | 40 |
| pET28C-6HIS-HsIQGAP1 (1-678) | MGSSHHHHHHSSGLVPRGSHMASMTGGQQMGRIPMSAADEVDGLGVARPHYGSVLDNERLTAEEMDERRRQNVAYEYLCHLEEAKRWMEACLGEDLPPTTELEEGLRNGVYLAKLGNFFSPKVVSLKKIYDREQTRYKATGLHFRHTDNVIQWLNAMDEIGLPKIFYPETTDIYDRKNMPRCIYCIHALSLYLFKLGLAPQIQDLYGKVDFTEEEINNMKTELEKYGIQMPAFSKIGGILANELSVDEAALHAAVIAINEAIDRRIPADTFAALKNPNAMLVNLEEPLASTYQDILYQAKQDKMTNAKNRTENSERERDVYEELLTQAEIQGNINKVNTFSALANIDLALEQGDALALFRALQSPALGLRGLQQQNSDWYLKQLLSDKQQKRQSGQTDPLQKEELQSGVDAANSAAQQYQRRLAAVALINAAIQKGVAEKTVLELMNPEAQLPQVYPFAADLYQKELATLQRQSPEHNLTHPELSVAVEMLSSVALINRALESGDVNTVWKQLSSSVTGLTNIEEENCQRYLDELMKLKAQAHAENNEFITWNDIQACVDHVNLVVQEEHERILAIGLINEALDEGDAQKTLQALQIPAAKLEGVLAEVAQHYQDTLIRAKREKAQEIQDESAVLWLDEIQGGIWQSNKDTQEAQKFALGIFAINEAVESGDVGKTLSALRSPDVGLYGVIP | 77 |
| pCDNA3-EGFP-GSGG-Rng2(1-189) | MVSKGEELFTGVVPILVELDGDVNGHKFSVSGEGEGDATYGKLTLKFICTTGKLPVPWPTLVTTLTYGVQCFSRYPDHMKQHDFFKSAMPEGYVQERTIFFKDDGNYKTRAEVKFEGDTLVNRIELKGIDFKEDGNILGHKLEYNYNSHNVYIMADKQKNGIKVNFKIRHNIEDGSVQLADHYQQNTPIGDGPVLLPDNHYLSTQSALSKDPNEKRDHMVLLEFVTAAGITLGMDELYKGSGGMDVNVGLSRLQSQAGAPVGTKGSNTRLAAKQRETLQAYDYLCRVDEAKKWIEECLGTDLGPTSTFEQSLRNGVVLALLVQKFQPDKLIKIFYSNELQFRHSDNINKFLDFIHGIGLPEIFHFELTDIYEGKNLPKVIYCIHALSYFLSMQDLAPPLIKSDENLSFTDEDVSIIVRRLRQSNVILPNFKA |  |
| pET28C-6HIS-Cdc12 (740-1391) | MGSSHHHHHHSSGLVPRGSHMASMTGGQQMGRIPGSTNSKERIIEYLLDKLDLRKKEIAAESTLWSNDGIDDKLRDLREQMSRQSSQPSTVSTILQIPDKKFHRPFPRHLHRYVGRSASESLTSEKDESIKSMKGIDDFANLEIPGKGIESNVVIKDISNQTHEINSVENKAETVSNNSKITNFDIPNDATSLPTIITHPTPPPPPPLPVKTSLNTFSHPDSVNIVANDTSVAGVMPAFPPPPPPPPPLVSAAGGKFVSPAVSNNISKDDLHKTTGLTRRPTRRLKQMHWEKLNSGLEFTFWTGPSDEANKILETLHTSGVLDELDESFAMKEAKTLVKKTCARTDYMSSELQKLFGIHFHKLSHKNPNEIIRMILHCDDSMNECVEFLSSDKVLNQPKLKADLEPYRIDWANGGDLVNSEKDASELSRWDYLYVRLIVDLGGYWNQRMNALKVKNIIETNYENLVRQTKLIGRAALELRDSKVFKGLLYLILYLGNYMNDYVRQAKGFAIGSLQRLPLIKNANNTKSLLHILDITIRKHFPQFDNFSPELSTVTEAAKLNIEAIEQECSELIRGCQNLQIDCDSGALSDPTVFHPDDKILSVILPWLMEGTKKMDFLKEHLRTMNTTLNNAMRYFGEQPNDPNSKNLFFKRVDSFIIDYSKARSDNLKSEEEEASQHRRLNLVNN | 77.5 |
| pETMCN-AScdc8 | MASMDKLREKINAARAETDEAVARAEAAEAKLKEVELQLSLKEQEYESLSRKSEAAESQLEELEEETKQLRLKADNEDIQKTEAEQLSRKVELLEEELETNDKLLRETTEKMRQTDVKAEHFERRVQSLERERDDMEQKLEEMTDKYTKVKAELDEVHQALEDL | 19.5 |
| pGEX4T1-GST-Fim1 | MSPILGYWKIKGLVQPTRLLLEYLEEKYEEHLYERDEGDKWRNKKFELGLEFPNLPYYIDGDVKLTQSMAIIRYIADKHNMLGGCPKERAEISMLEGAVLDIRYGVSRIAYSKDFETLKVDFLSKLPEMLKMFEDRLCHKTYLNGDHVTHPDFMLYDALDVVLYMDPMCLDAFPKLVCFKKRIEAIPQIDKYLKSSKYIAWPLQGWQATFGGGDHPPKSDLVPRGSMLALKLQKKYPELTNEEILTLTDQFNKLDVDGKGYLDQPTTIKAFEDSKKGSYDEVREAIREVNVDSSGRVEPEDFVGIFNVLKKGVEGTEVKKGRITIKGSSSSVSHTINEEERREFIKHINSVLAGDPDVGSRVPINTETFEFFDQCKDGLILSKLINDSVPDTIDERVLNKQRNNKPLDNFKCIENNNVVINSAKAMGGISITNIGAGDILEGREHLILGLVWQIIRRGLLGKIDITLHPELYRLLEEDETLDQFLRLPPEKILLRWFNYHLKAANWPRTVSNFSKDVSDGENYTVLLNQLAPELCSRAPLQTTDVLQRAEQVLQNAEKLDCRKYLTPTAMVAGNPKLNLAFVAHLFNTHPGLEPLNEEEKPEIEPFDAEGEREARVFTLWLNSLDVTPSIHDFFNNLRDGLILLQAYDKITPNTVNWKKVNKAPASGDEMMRFKAVENCNYAVDLGKNQGFSLVGIQGADITDGSRTLTLALVWQMMRMNITKTLHSLSRGGKTLSDSDMVAWANSMAAKGGKGSQIRSFRDPSISTGVFVLDVLHGIKSEYVDYNLVTDGSTEELAIQNARLAISIARKLGAVIFILPEDIVAVRPRLVLHFIGSLMAV | 94.5 |
| pET23a-10HIS-SNAP-Ezrin-ABD | MGSSHHHHHHHHHHSSGLVPRGSHMASMTGGQQMGRGSDKDCEMKRTTLDSPLGKLELSGCEQGLHRIIFLGKGTSAADAVEVPAPAAVLGGPEPLMQATAWLNAYFHQPEAIEEFPVPALHHPVFQQESFTRQVLWKLLKVVKFGEVISYSHLAALAGNPAATAAVKTALSGNPVPILIPCHRVVQGDLDVGGYEGGLAVKEWLLAHEGHRLGKPGLGGSGSGSGSGGGGSSTSVYEPVSYHVQESLQDEGAEPTGYSAELSSEGIRDDRNEEKRITEAEKNERVQRQLLTLSSELSQARDENKRTHNDIIHNENMRQGRDKYKTLRQIRQGNTKQRIDEFEAL | 37.5 |
