# Supplementary material for: Calponin-homology domain mediated bending of membrane-associated actin filaments
Source: eLife. 2021 Jul 16;10:e61078. doi: 10.7554/eLife.61078 (PMC8315802; doi:10.7554/eLife.61078)
Supplement: Transparent reporting form [file elife-61078-transrepform.docx]

***eLife’s* transparent reporting form**

We encourage authors to provide detailed information *within their submission* to facilitate the interpretation and replication of experiments. Authors can upload supporting documentation to indicate the use of appropriate reporting guidelines for health-related research (see [EQUATOR Network](http://www.equator-network.org/%20)), life science research (see the [BioSharing Information Resource](https://biosharing.org/)), or the [ARRIVE guidelines](http://www.plosbiology.org/article/info:doi/10.1371/journal.pbio.1000412) for reporting work involving animal research. Where applicable, authors should refer to any relevant reporting standards documents in this form.

If you have any questions, please consult our Journal Policies and/or contact us: [editorial@elifesciences.org](mailto:editorial@elifesciences.org).

**Sample-size estimation**

- You should state whether an appropriate sample size was computed when the study was being designed
- You should state the statistical method of sample size computation and any required assumptions
- If no explicit power analysis was used, you should describe how you decided what sample (replicate) size (number) to use

Please outline where this information can be found within the submission (e.g., sections or figure legends), or explain why this information doesn’t apply to your submission:

Our experiments involved purified proteins. For each condition, we usually performed at least 3 independent experiments each generating tens to hundreds of individual protein-protein interactions sufficient to conclude an effect. For each condition or day of experiment, we performed in parallel a control experiment with His6-Rng2(1-189) to ensure that the system was behaving normal. We did not explicitly compute sample sizes while designing the study because the available material was not a limiting factor and aimed (based on experience) to obtain >50 measurements for each condition.

For experiments with mammalian cell lines, we performed two independent experiments for each condition and imaged usually 10 randomly selected cells.

**Replicates**

- You should report how often each experiment was performed
- You should include a definition of biological versus technical replication
- The data obtained should be provided and sufficient information should be provided to indicate the number of independent biological and/or technical replicates
- If you encountered any outliers, you should describe how these were handled
- Criteria for exclusion/inclusion of data should be clearly stated
- High-throughput sequence data should be uploaded before submission, with a private link for reviewers provided (these are available from both GEO and ArrayExpress)

Please outline where this information can be found within the submission (e.g., sections or figure legends), or explain why this information doesn’t apply to your submission:

The number of independent experiments and number of measurements are indicated in the text and in the figure legends for each parameter that was quantified in the study. We performed at least two independent experiments and obtained >50 measurements (actin filament curvature measurement) for each condition (except for one construct that showed very weak binding).

The number N of measurements indicates biological replicates as each curvature measurement was performed on another actin filaments.

We did not perform technical replicates as we assumed that the biological variance would be much larger than the technical variance.

We did not exclude outliers in the computation of the average and the standard deviation.

All graphs display the data distribution, mean, median and SD.

**Statistical reporting**

- Statistical analysis methods should be described and justified
- Raw data should be presented in figures whenever informative to do so (typically when N per group is less than 10)
- For each experiment, you should identify the statistical tests used, exact values of N, definitions of center, methods of multiple test correction, and dispersion and precision measures (e.g., mean, median, SD, SEM, confidence intervals; and, for the major substantive results, a measure of effect size (e.g., Pearson's r, Cohen's d)
- Report exact p-values wherever possible alongside the summary statistics and 95% confidence intervals. These should be reported for all key questions and not only when the p-value is less than 0.05.

Please outline where this information can be found within the submission (e.g., sections or figure legends), or explain why this information doesn’t apply to your submission:

The number of independent experiments and number of measurements are indicated in the text and in the figure legends for each parameter that was quantified in the study.

We did not exclude outliers in the computation of the average and the standard deviation.

All graphs display the data distribution, mean, median and SD. And we added tables depicting the descriptive statistics as well as the ANOVA – Tukey means comparison in Supplementary File 1.

(For large datasets, or papers with a very large number of statistical tests, you may upload a single table file with tests, Ns, etc., with reference to sections in the manuscript.)

**Group allocation**

- Indicate how samples were allocated into experimental groups (in the case of clinical studies, please specify allocation to treatment method); if randomization was used, please also state if restricted randomization was applied
- Indicate if masking was used during group allocation, data collection and/or data analysis

Please outline where this information can be found within the submission (e.g., sections or figure legends), or explain why this information doesn’t apply to your submission:

Since this is a study of protein-protein interactions using purified proteins, groups were based on the different proteins used in each experiment.

Masking was not used during group allocation, data collection or data analysis as the difference between conditions (actin ring formation or not) was obvious.

**Additional data files (“source data”)**

- We encourage you to upload relevant additional data files, such as numerical data that are represented as a graph in a figure, or as a summary table
- Where provided, these should be in the most useful format, and they can be uploaded as “Source data” files linked to a main figure or table
- Include model definition files including the full list of parameters used
- Include code used for data analysis (e.g., R, MatLab)
- Avoid stating that data files are “available upon request”

Please indicate the figures or tables for which source data files have been provided:

We uploaded all raw images used for the curvature analysis on dryad and provided a summary table of the curvature measurements in Supplementary File 1.
